# Giant second part of duodenal perforation: Case report and management challenge

**DOI:** 10.1016/j.ijscr.2025.111521

**Published:** 2025-06-14

**Authors:** Atalel Fentahun Awedew, Kidist Hunegn Setargew, Andualem Dagne Tebkew, Zemen Asmare Emiru

**Affiliations:** a Department of Surgery, SOM, Debre Tabor University, Debre Tabor, Ethiopia; b Department of Surgery, SOM, Bahir Dar University, Bahir Dar, Ethiopia

**Keywords:** Duodenal perforation, PUD, Ethiopia, Case report

## Abstract

**Introduction and importance:**

Duodenal perforation poses a significant global health challenge, contributing substantially to morbidity, mortality, and hospitalizations. While most perforations occur in the first part of the duodenum, are typically small, and affect older individuals, giant perforations in the second part of the duodenum are exceedingly rare.

**Case presentation:**

A 20-year-old male patient presented with a three-day history of diffuse abdominal pain, accompanied by nausea, vomiting, and anorexia of similar duration. The physical examination revealed PR = 104, BP = 110/70 mmHg and diffuse abdominal tenderness. A significant intraoperative finding was a 5 × 6 cm perforation in the second portion of the duodenum, with leakage of gastrointestinal and biliary contents consistent with biliary peritonitis. A pyloric exclusion, retrocolic gastrojejunostomy, and duodenal repair were performed.

**Clinical discussion:**

Perforations in the second part of the duodenum are considerably rarer. However, when perforation does occur, particularly in the second part of the duodenum, management presents significant challenges, often associated with high rates of morbidity and mortality. Our patient presented with one the high-risk factors—delayed presentation—and, based on established risk stratification models, would therefore carry an estimated mortality risk of approximately 10 %. There is currently a paucity of definitive guidelines or strong expert consensus regarding the optimal surgical approach for giant duodenal perforations located in the second part. In our patient's case, we performed a repair of the perforation, pyloric exclusion, and retrocolic gastrojejunostomy.

**Conclusion:**

Perforation of the second part of the duodenum is a rare occurrence and presents significant management challenges in emergency situations.

## Highlights

• Giant duodenal perforation in second part is rare and had sever morbidity and mortality
• Giant duodenal perforation in second part management is challenging and no standard surgical recommendation
• Management of giant duodenal perforation in this case was aggressive resuscitation, pyloric exclusion, retrocolic gastrojejunostomy, duodenal repair, antibiotics with support and eradication therapy and long term PPI course.

## 1 Introduction

**Fig. 1:**
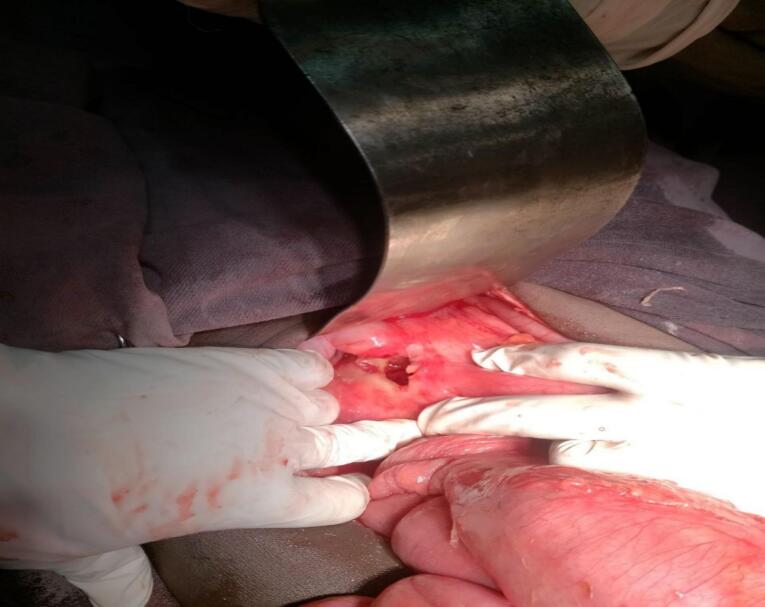
Giant duodenum perforation in second part of duodenum part (5*6 cm).

**Fig. 2:**
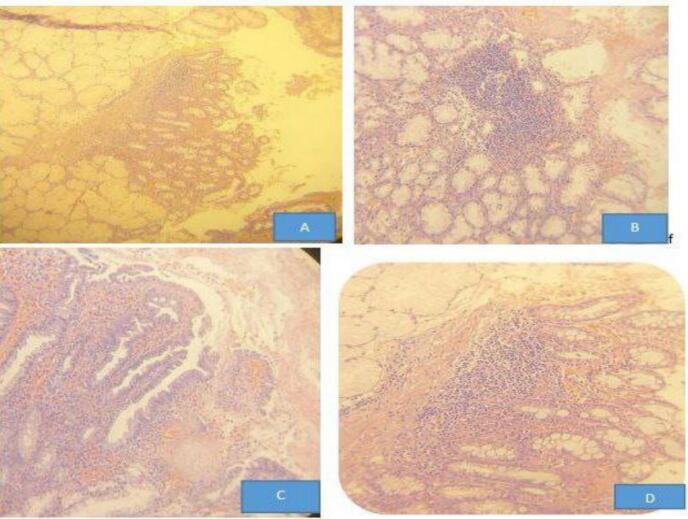
Histologic sections through duodenal tissues show A. Brunner gland hyperplasia with evidence of glandular aggregate above the muscularis mucosa B. Chronic inflammatory cell predominantly lymphocytic aggregates in duodenal epithelium C. Focal areas of foveolar metaplasia of duodenal surface epithelium D. Intraepithelial mixed inflammatory cell predominantly lymphocytic infiltrate.

Peptic ulcer disease (PUD) is characterized by a breach in the mucosal lining of the gastrointestinal tract, typically exceeding 3–5 mm in diameter, and extending into the submucosa [1,2]. These ulcers predominantly occur in the stomach and proximal duodenum. PUD represents a significant global health concern, contributing substantially to morbidity, mortality, and hospital admissions [3] While the estimated lifetime prevalence of PUD is approximately 5–10 % [2,4], with an annual incidence of 0.1–0.3 % in general populations within Western countries, the global prevalence in 2019 was approximately 8.09 million [3]. Despite this high prevalence, age-standardized incidence, mortality, and disability-adjusted life years (DALYs) associated with PUD have demonstrated a decline over the past three decades, largely due to the widespread adoption of *Helicobacter pylori* eradication therapies and the introduction of proton pump inhibitors (PPIs) [3,5]. PUD can manifest with several complications, including perforation, bleeding, and gastric outlet obstruction [6,7]. Perforation, specifically, tends to occur most frequently in the stomach or the first part of the duodenum. Acute perforations of the duodenum are estimated to occur in 2–10 % of patients with duodenal ulcers [7]. These perforations are classified based on their size as small, large, or giant, although a globally accepted classification system has not been established. The standard surgical approach for small duodenal ulcer perforations involves the use of a pedicled omental patch, originally described by Cellan-Jones in 1921 [8], or a free omental graft, described by Graham in 1937 [9], with either exhibiting equivalent efficacy in outcomes. These techniques involve drawing a strand of omentum over the perforation site and securing it in place with full-thickness sutures positioned on either side of the perforation [8,9]. (See Fig. 1, Fig. 2.)

However, the optimal surgical management of large and giant perforations in the first part of the duodenum remains controversial, with proposed approaches including partial gastrectomy, jejunal serosal patching, and other more complex surgical procedures. Furthermore, there is a scarcity of evidence in the literature regarding the management of giant perforations in the second part of the duodenum. This case report contributes to addressing this knowledge gap by providing detailed procedural approaches and outcomes of a case involving a giant second-part duodenal perforation with associated biliary peritonitis. This case report adheres to the SCARE 2025 guidelines for reporting surgical case reports [10].

## 2 Case presentation

A 20-year-old male patient presented with a three-day history of diffuse abdominal pain, accompanied by nausea, vomiting, and anorexia of similar duration. He also reported a three-month history of dyspepsia. The physical examination revealed an alert and well-nourished individual with a pulse rate of 104 beats per minute and a blood pressure of 110/70 mmHg. Diffuse abdominal tenderness was present in all quadrants; however, the cardiovascular and respiratory examinations were unremarkable. Initial laboratory investigations included a complete blood count (CBC), which showed a white blood cell count (WBC) of 11,000/μL with 87 % neutrophils; other blood parameters were within normal limits. An abdominal ultrasound suggested the presence of an intra-abdominal abscess and a perforated appendix.

Following fluid resuscitation with two liters of normal saline, exploratory laparotomy was performed. Intraoperative findings revealed approximately three liters of purulent fluid with minimal bile. A significant finding was a 5 × 6 cm perforation in the second portion of the duodenum, with leakage of gastrointestinal and biliary contents consistent with biliary peritonitis. The patient remained hemodynamically stable throughout the procedure. The abdomen was copiously irrigated with warm saline solution, and tissue biopsies were obtained. A pyloric exclusion, retrocolic gastrojejunostomy, and kocherization of the duodenum and repair were performed. A subhepatic drain was placed.

The nasogastric tube (NGT) was removed after 24 h, and the patient was started on sips of fluids after 48 h. The subhepatic drain showed no output and was removed after 72 h. The postoperative course was uneventful. The patient received *Helicobacter pylori* eradication therapy and a three-month course of proton pump inhibitors (PPIs). At follow-up, the patient was doing well. Histopathological examination of the biopsy revealed chronic inflammation consistent with perforated peptic ulcer disease.

## 3 Discussion

Duodenal ulceration has significantly declined in incidence, largely attributed to the widespread use of proton pump inhibitors (PPIs) and the effective eradication of *Helicobacter pylori* [[1], [2], [3]]. While duodenal ulcers still occur, they are most commonly located in the first part of the duodenum and are more frequently observed in older individuals. Perforations in the second part of the duodenum are considerably rarer, with occurrences in young adults being exceptionally uncommon [11]. However, when perforation does occur, particularly in the second part of the duodenum, management presents significant challenges, often associated with high rates of morbidity and mortality [11]. Several risk scoring systems have been developed to predict outcomes in patients with perforated peptic ulcers [12].

Boey et al. identified preoperative shock, significant comorbid conditions, and delayed presentation as major prognostic factors influencing postoperative mortality [12]. This scoring system predicted postoperative mortality as follows: one factor presents, 10 % mortality; two factors, 45.5 % mortality; and three factors, 100 % mortality. Our patient presented with one of these high-risk factors—delayed presentation—and, based on Boey et al.'s risk stratification model, would therefore carry an estimated mortality risk of approximately 10 %. Management strategies are tailored to the individual patient, taking into account hemodynamic status, comorbidities, and overall fitness for extensive surgical procedures. The principles of damage control surgery, which prioritize stabilization and resuscitation before definitive repair, are frequently employed in patients deemed unfit for immediate extensive surgery at the time of presentation. This approach allows for staged interventions, reducing risks and improving outcomes in high-risk individuals.

The management of small duodenal perforations, and in some cases larger perforations, often involves surgical intervention. The classic pedicled omental patch, a technique used to ‘plug’ these perforations, was first described by Cellan-Jones in 1929 [8]. While commonly and incorrectly attributed to Graham, who detailed the use of a free omental graft for perforation repair in 1937 [9], the pedicled omental patch involves drawing a strand of omentum over the perforation, securing it with full-thickness sutures placed on either side of the defect. This technique has become the gold standard for the treatment of small duodenal perforations. For large and giant perforations of the first part of the duodenum, most guidelines and authors recommend partial gastrectomy with reconstruction via either a Billroth I or Billroth II anastomosis [13]. More complex procedures, such as gastric disconnection, involving vagotomy, antrectomy, gastrostomy, lateral duodenostomy, and feeding jejunostomy, with subsequent elective restoration of intestinal continuity after approximately four weeks, are sometimes required for especially complex cases [13].

Other surgical options suggested by various guidelines and authors for the management of giant or large perforations include conversion of the perforation into a pyloroplasty; closure of the perforation using a serosal patch or a pedicled graft of the jejunum; or a free omental plug, combined with a proximal gastrojejunostomy and/or vagotomy, designed to divert gastric contents and to achieve definitive acid reduction, respectively [13].

One study reported on two cases of ‘giant’ first-part duodenal perforations (defined as more than 3 cm in size). One case was treated with antrectomy and Billroth II reconstruction, while the other underwent a jejunal serosal patch repair. The patient undergoing antrectomy, unfortunately, succumbed to septicemia on the first postoperative day, whereas the patient who received a jejunal serosal patch survived [13]. It's important to note that there is currently a paucity of definitive guidelines or strong expert consensus regarding the optimal surgical approach for giant duodenal perforations located in the second part. In our patient's case, we performed a repair of the perforation, pyloric exclusion, and retrocolic gastrojejunostomy. The rationale behind this approach was to reduce gastrointestinal content flow into the duodenum and divert it to the jejunum, thereby promoting duodenal healing. We found a case report in which a 57-year-old patient with comorbidities and shock was managed with a triple-ostomy procedure as a primary intervention to reduce operative time and to avoid potential leakage from the repair site [11]. Given that our patient was young, hemodynamically stable both pre- and intraoperatively, and without significant comorbidities, we were able to proceed with the more complex surgical procedure described above. Repair, pyloric exclusion, and gastrojejunostomy are primarily employed in the management of severe traumatic duodenal injuries [14]. A simplified ‘diverticulization’ technique has been developed that obviates the need for gastric resection [14]. This procedure temporarily redirects gastrointestinal contents away from the duodenum, thus affording protection during the initial postoperative healing phase while facilitating the eventual restoration of normal duodenal function [14].

In addition to surgical intervention, *Helicobacter pylori* (*H. pylori*) eradication therapy and long-term proton pump inhibitor (PPI) therapy were essential components of the standard management approach for complicated peptic ulcer disease (PUD), aiding in ulcer healing and preventing recurrence.

## 4 Conclusion

Giant perforations of the second part of the duodenum are rare occurrences, presenting significant challenges in management and associated with high mortality and morbidity rates. One effective approach to managing these complex cases is a strategy that incorporates kocherization of the duodenum, primary repair, pyloric exclusion, and gastrojejunostomy. This technique, guided by the principle of diverticulization, avoids gastric resection. By temporarily redirecting gastrointestinal contents away from the duodenum, it provides protection during the initial postoperative healing period, ultimately facilitating the restoration of normal duodenal function.

## Consent for publication

Written informed consent was obtained from the patient for publication and any accompanying images. A copy of the written consent is available for review by the Editor-in-Chief of this journal on request.

## Ethical approval and consent to participate

Written informed consent was obtained from the patient for publication and any accompanying images and exempt from ethical approval when the patient provides consent or a guarantee. A copy of the consent is available upon request.

## Declaration of Generative AI and AI-assisted technologies in the writing process

Language model AI was employed to improve the grammer and spelling checking during manuscript writing.

## Funding

Not applicable.

## Declaration of competing interest

There are no reported conflicts of interest for the writers.

## Data Availability

Not applicable.
